# Increase in imported mupirocin-resistant *Staphylococcus aureus* from the tropics and subtropics: trends in Berlin from 2018 to 2025

**DOI:** 10.1016/j.nmni.2026.101740

**Published:** 2026-03-25

**Authors:** Maria Cristina Moreno-del Castillo, Daniel Humme, Rasmus Leistner, Renate Krüger, Miriam Stegemann, Leif Hanitsch, Franziska Layer-Nicolaou, Andreas Knaust, Josef G. Sägmüller, Angela Stein, Paul Pitzinger, Alice Hucko, Jessica L. Rohmann, Frank P. Mockenhaupt, Beate Kampmann, Dennis Nurjadi, Philipp Zanger, Andreas K. Lindner

**Affiliations:** a Charité - Universitätsmedizin Berlin, Charité Center for Global Health, Institute of International Health, Berlin, Germany; b Charité - Universitätsmedizin Berlin, Interdisciplinary Workgroup on PVL-positive S. aureus, Berlin, Germany^1^; c Charité-Universitätsmedizin Berlin, Department of Dermatology, Venereology and Allergology, Berlin, Germany; d Charité - Universitätsmedizin Berlin, Department of Gastroenterology, Infectious Diseases, and Rheumatology, Berlin, Germany; e Charité – Universitätsmedizin Berlin, Department of Pediatric Respiratory Medicine, Immunology and Critical Care Medicine, Berlin, Germany; f Charité-Universitätsmedizin Berlin, Department of Infectious Diseases and Critical Care Medicine, Berlin, Germany; g Charité-Universitätsmedizin Berlin, Universitätsmedizin Berlin, Institute of Medical Immunology, Berlin, Germany; h Division Nosocomial Pathogens and Antibiotic Resistances, Department of Infectious Diseases, National Reference Centre for Staphylococci and Enterococci, Robert Koch Institute, Wernigerode Branch, Wernigerode, Germany; i Labor Berlin Charité Vivantes GmbH, Berlin, Germany; j Berlin Institute of Health at Charité- Universitätsmedizin Berlin, QUEST Center for Responsible Research, Berlin, Germany; k University of Lübeck and University Hospital Schleswig-Holstein, Lübeck, Institute of Medical Microbiology, Lübeck, Germany; l German Center for Infection Research, Hamburg-Lübeck-Borstel-Riems site, Lübeck, Germany; m University Hospitals, Heidelberg, Heidelberg Institute of Global Health (HIGH), Heidelberg, Germany

**Keywords:** *Staphylococcus aureus*, Mupirocin resistance, Antimicrobial resistance, Decolonisation, Panton-valentine leucocidin, PVL, Skin and soft tissue infections, Methicillin-resistant *Staphylococcus aureus*, MRSA, Infection control

## Abstract

**Background:**

Mupirocin resistance in *Staphylococcus (S.) aureus* (Mup-RSA) is emerging and may lead to *S. aureus* decolonisation failure. Our aim was to assess temporal trends in mupirocin-resistant *S. aureus* (Mup-RSA) and methicillin-resistant *S. aureus* (MRSA) among *S. aureus*–positive patients presenting to the Tropical Medicine outpatient Department, also including the Dermatology Department and the overall university hospital for context.

**Methods:**

We calculated annual percentages of Mup-RSA and MRSA among *S. aureus*–positive patients and modelled temporal trends using Poisson or negative binomial regression, estimating incidence rate ratios (IRR).

**Results:**

From January 1, 2018, to June 30, 2025, Mup-RSA was detected in 3.1% (24/782) of patients with *S. aureus* at the Tropical Medicine Department, 1.1% (23/2030) at the Dermatology, and 0.5% (142/28,122) at the hospital overall. The rate of Mup-RSA among *S. aureus*-positive patients increased over time at the Tropical Medicine (IRR, 1.64; 95% CI, 1.3 – 2.06, *p < 0.01*), and at the Dermatology (IRR, 1.37; 95% CI, 1.12 – 1.67, *p < 0.01*), but not at the hospital overall. MRSA did not increase significantly over time in either setting. MRSA in Mup-RSA was found in 4.2% (1/24), 4.3% (1/23), and 22.5% (32/142), respectively.

**Conclusion:**

Mup-RSA proportions are low overall, but have increased substantially in Tropical Medicine, with modest increases in Dermatology. Clinicians should be aware of both the emergence of Mup-RSA and the availability of alternative topical agents for decolonisation, particularly in contexts where *S. aureus* could be imported from regions with high Mup-RSA prevalence.

## Highlights

• Mupirocin-resistant *Staphylococcus aureus* is emerging in tropical medicine outpatients.
• Imported cases in Berlin identified since 2020; mainly after travel to Southeast Asia.
• Among *S. aureus*-positive patients, resistance rates highest (9.2%) in first half of 2025.
• Smaller increases in Dermatology Department; overall hospital rates stayed stable.
• Consider mupirocin susceptibility testing, esp. after travel or decolonisation failure.

## 1 Introduction

Mupirocin is an antibiotic nasal ointment for *Staphylococcus (S.) aureus* used as topical treatment for skin and soft tissue infections (SSTIs), and as part of *S. aureus* decolonisation protocols. Decolonisation protocols are indicated for prevention of recurrence and transmission in patients infected with or carrying methicillin-resistant *S. aureus* (MRSA) [1,2], as well as in patients with Panton-Valentine Leucocidin (PVL) positive *S. aureus* infections [3,4]. Mupirocin resistance (Mup-R) in *S. aureus* (Mup-RSA) is an emerging threat that has been previously implicated with decolonisation failure [5]. Given the lack of alternatives for specific nasal decolonisation, the emergence of Mup-R poses a threat to both effective clinical care and public health containment strategies [[5], [6], [7], [8], [9]]. Unfortunately, in this regard, Mup-RSA frequently remains underrecognized in clinical practice, in part because awareness is low. Two main mechanisms of mupirocin resistance (Mup-R) have been described. The predominant mechanism is acquisition of the *mupA* gene, located on a conjugative plasmid, which confers high-level Mup-R, defined by a minimal inhibitory concentration (MIC) exceeding 512 mg/L [[10], [11], [12]]. A second mechanism involves point mutations in the native *ileS* gene, resulting in low-level Mup-R with more variable and less well-understood clinical implications [10,13]. While the *mupA gene* does not encode resistance to other antibiotics, *mupA*-carrying plasmids have been associated with co-resistance to other antimicrobial classes (e.g., erythromycin, clindamycin, levofloxacin, and tetracycline), suggesting that plasmids may carry additional resistance genes [11]. In Germany, the Commission for Hospital Hygiene and Infection Prevention (KRINKO) recommends mupirocin as the agent of choice for nasal decolonisation in MRSA carriers, provided there is no resistance or previous treatment failure [2]. Similarly, the latest 2011 Infectious Diseases Society of America (IDSA) guidelines support mupirocin-based nasal decolonisation, typically for 5–10 days, as part of a broader approach for patients with recurrent SSTIs or ongoing transmission despite optimized hygiene [1]. In both settings, mupirocin is frequently used in combination with topical antiseptic regimens such as chlorhexidine, especially in high-risk or recurrent cases [1,2].

The study was initiated after clinical observations of decolonisation failure associated with Mup-RSA suggested a potentially emerging problem. Our primary aim was to assess temporal trends in Mup-RSA and MRSA among patients with *S. aureus* positive purulent SSTIs seen from 2018 through June 2025 at the Tropical Medicine outpatient department (OPD). Our secondary aim was to assess temporal trends of Mup-RSA and MRSA among patients with *S. aureus* evaluated at the local Dermatology Department, and the University hospital overall during the same time-period, as contextual comparators to the Tropical Medicine OPD population.

## 2 Methods

### 2.1 Ethics statement

The study received ethical approval for use of routinely collected data from the ethics committee of Charité–Universitätsmedizin Berlin (EA2/260/25). Patient consent was not required for the retrospective evaluation of routine clinical data in accordance with the Berlin State Hospital Act.

### 2.2 Study population and data sources

We included all *S. aureus*–positive patients, that were identified between January 1, 2018, and June 30, 2025. Patients were tested for diagnostic or screening purposes at the discretion of the attending physicians following institutional protocols. Microbiological data were retrospectively obtained from the central reference laboratory. Data were extracted from the laboratory information system. To avoid duplicates, we included the number of patients per year with at least one sample positive for *S. aureus*, and proportions of high-level Mup-RSA and MRSA were calculated at the patient level. Data was stratified for the following sites: (1) the subgroup of patients from the Tropical Medicine OPD, which offers care for returning travellers, migrants, and refugees and serves as a referral centre for imported infections; (2) the subgroup of patients from the Dermatology Department, which provides specialized care for patients with complex dermatologic and allergic conditions, (3) all patients identified at the University Hospital, a large academic medical centre providing tertiary outpatient and inpatient care in Berlin with over 3000 inpatient beds. The study was conducted in cooperation with *StaphTrav*, a European surveillance network for imported *S. aureus*-positive purulent SSTIs [14].

### 2.3 Microbiological evaluation

Clinical specimens were obtained using Copan swabs with Amies transport medium and streaked onto non-selective and selective agar media for *S. aureus* (BD). Cultures were incubated for 24–48 h at 36 °C in an atmosphere containing 5 % CO_2_. After incubation, plates were read, and β-haemolytic colonies were sub-cultured. Species identification was performed using matrix-assisted laser desorption/ionization time-of-flight mass spectrometry (MALDI-TOF MS; Bruker).

Antimicrobial susceptibility testing was carried out using the automated VITEK® 2 system (bioMérieux). High-level Mup-R was defined as a MIC >512 mg/L [12]. Methicillin resistance was determined based on concomitant resistance to both oxacillin and cefoxitin, which was required for phenotypic classification as MRSA. All MRSA isolates underwent additional confirmation using a rapid MRSA detection assay (Coris BioConcept). In cases of discordant phenotypic results, molecular confirmation was performed by detection of the *mecA* and/or *mecC* resistance genes using a PCR-based line probe assay with reverse hybridization (GenoType MRSA, Hain Lifescience GmbH).

### 2.4 Statistical analysis

We calculated the proportions per year of Mup-RSA and MRSA among *S. aureus* positive patients out of total patients tested from 2018 to 2025 in the Tropical Medicine OPD, the Dermatology department, and the overall University hospital. 95% confidence intervals (CIs) for proportions were constructed using Wilson score intervals, as recommended for small sample sizes, assuming independent binomial probability distributions [15]. Proportions are expressed as percentages. Graphs were created to visualize annual Mup-RSA and MRSA proportion changes over the years.

We used Poisson regression to model annual counts of high-level Mup-RSA and MRSA and assess temporal trends, fitting separate models for the Tropical Medicine OPD, the Dermatology Department, and the overall University hospital. The dependent variable was the annual count of patients with at least one resistant *S. aureus* isolate; the logarithm of the annual number of patients with *S. aureus* isolated was included as an offset, and calendar year was included as a continuous predictor. Robust standard errors (heteroskedasticity-consistent, HC3) were applied to obtain conservative variance estimates appropriate for small samples [16]. Model fit was evaluated using Pearson residuals and the dispersion parameter to assess overdispersion. Models demonstrating substantial overdispersion were refitted using negative binomial regression. Results from the models are expressed as incidence rate ratios (IRR) with 95% CIs. IRRs >1 indicate increasing resistance by calendar year, with higher values reflecting a larger annual increase. A two-tailed *p-value* < 0.05 was considered statistically significant. Data analyses were conducted in R (version 4.5.1; R Core Team) using the packages *tidyverse*, *dplyr*, *readxl*, *binom, DescTools*, *MASS*, *sandwich,* and *ggrepel* [[17], [18], [19], [20], [21], [22], [23], [24], [25]].

## 3 Results

**Fig. 1:**
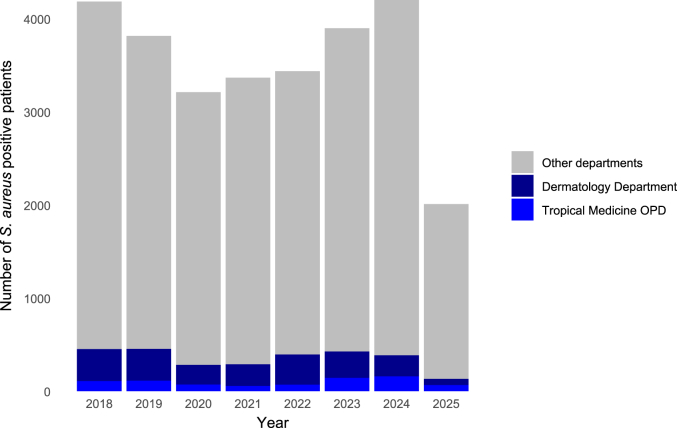
Annual number of *S. aureus*–positive patients by clinical setting (2018 – 2025) Annual number of patients with at least one *S. aureus*–positive culture at the University hospital in Berlin, stratified by clinical setting. Data for 2025 reflect the first half of the calendar year. Abbreviations: *S. aureus*, *Staphylococcus aureus*; OPD, outpatient department.

Between 2018 and June 2025, a total of 28,122 *S. aureus*-positive patients were identified at the overall University hospital. Of the overall patients, the Tropical Medicine OPD contributed 782 (2.8%), and the Dermatology Department contributed 2030 (7.2%) patients; see Fig. 1.

### 3.1 Tropical medicine outpatient department

**Fig. 2:**
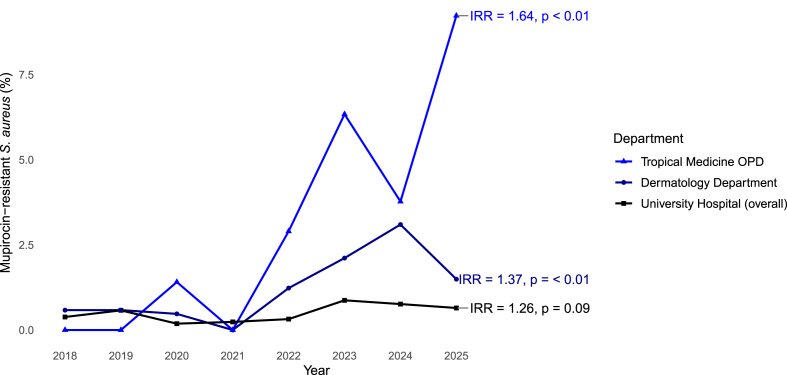
**Temporal trends in the percentage of Mup-RSA among patients with *S. aureus* by clinical setting (2018**–**2025)** Annual percentage of patients with Mup-RSA at the University Hospital in Berlin, stratified by clinical setting. IRRs and corresponding *p values* from regression models assessing temporal increases are displayed at the end of each series. Data for 2025 reflect the first half of the calendar year. Abbreviations: MupRSA, mupirocin-resistant *Staphylococcus aureus*; *S. aureus*, *Staphylococcus aureus*; OPD, outpatient department; IRRs, incidence risk ratios.

Mup-RSA was seen in a total of 24/782 patients (3.1%) at the Tropical Medicine OPD during the full study period. Mup-RSA was first detected in 2020, with the highest number of cases in the first half of 2025 (9.2%); see Table 1. Each one-year increase corresponded to a 64% higher rate of Mup-R cases per patient with *S. aureus* isolated (IRR, 1.64; 95% CI, 1.3 – 2.06, *p < 0.01*) obtained from Poisson regression, see Fig. 2.

**Table 1:** Mupirocin and methicillin resistance in *S. aureus* by year by clinical settings in Berlin between January 2018 and June 2025.

Year	*S. aureus*		Mup-RSA		MRSA
	n	n	% (95% CI)	n	% (95% CI)
***Tropical Medicine OPD***					
2018	108	0	0.0% (0.0 – 3.4%)	11	10.2% (5.8 – 17.3%)
2019	112	0	0.0% (0.0 – 3.3%)	16	14.3% (9.0 – 22%)
2020	71	1	1.4% (0.2 – 7.6%)	5	7.0% (3.0 – 15.4%)
2021	56	0	0.0% (0.0 – 6.4%)	9	16.1% (8.7 – 27.8%)
2022	69	2	2.9% (0.8 – 10%)	11	15.9% (9.1 – 26.3%)
2023	142	9	6.3% (3.4 – 11.6%)	26	18.3% (12.8 – 25.5%)
2024	159	6	3.8% (1.7 – 8%)	21	13.2% (8.8 – 19.3%)
a2025	65	6	9.2% (4.3 – 18.7%)	6	9.2% (4.3 – 18.7%)
***Dermatology Department***					
2018	343	2	0.6% (0.2 – 2.1%)	19	5.5% (3.6 – 8.5%)
2019	342	2	0.6% (0.2 – 2.1%)	14	4.1% (2.5 – 6.8%)
2020	211	1	0.5% (0.1 – 2.6%)	21	10.0% (6.6 – 14.7%)
2021	232	0	0.0% (0.0 – 1.6%)	17	7.3% (4.6 – 11.4%)
2022	325	4	1.2% (0.5 – 3.1%)	32	9.8% (7.1 – 13.6%)
2023	284	6	2.1% (1.0 – 4.5%)	32	11.3% (8.1 – 15.5%)
2024	226	7	3.1% (1.5 – 6.3%)	16	7.1% (4.4 – 11.2%)
a2025	67	1	1.5% (0.3 – 8%)	6	9.0% (4.2 – 18.2%)
***University Hospital (overall)***					
2018	4184	16	0.4% (0.2 – 0.6%)	538	12.9% (11.9 – 13.9%)
2019	3815	22	0.6% (0.4 – 0.9%)	459	12.0% (11.0 – 13.1%)
2020	3211	6	0.2% (0.1 – 0.4%)	351	10.9% (9.9 – 12.1%)
2021	3367	8	0.2% (0.1 – 0.5%)	336	10.0% (9.0 – 11.0%)
2022	3437	11	0.3% (0.2 – 0.6%)	395	11.5% (10.5 – 12.6%)
2023	3898	34	0.9% (0.6 – 1.2%)	443	11.4% (10.4 – 12.4%)
2024	4201	32	0.8% (0.5 – 1.1%)	514	12.2% (11.3 – 13.3%)
a2025	2009	13	0.6% (0.4 – 1.1%)	251	12.5% (11.1 – 14%)

Mupirocin-resistant and methicillin-resistant *S. aureus* percentages at the University hospital overall and in the subgroup of patients evaluated at the Tropical Medicine OPD and Dermatology Department. Percentages are calculated using the annual number of *S. aureus*–positive patients as the denominator for each setting. The “University Hospital (overall)” category includes patients seen in the Dermatology and Tropical Medicine outpatient clinics as well as all other hospital departments.

Abbreviations: *S. aureus*, *Staphylococcus aureus*; Mup-RSA, mupirocin-resistant *S. aureus*; MRSA, methicillin-resistant *S. aureus*; CI, confidence interval; OPD, outpatient department.

a From January 1st to June 30th, 2025.

**Fig. 3:**
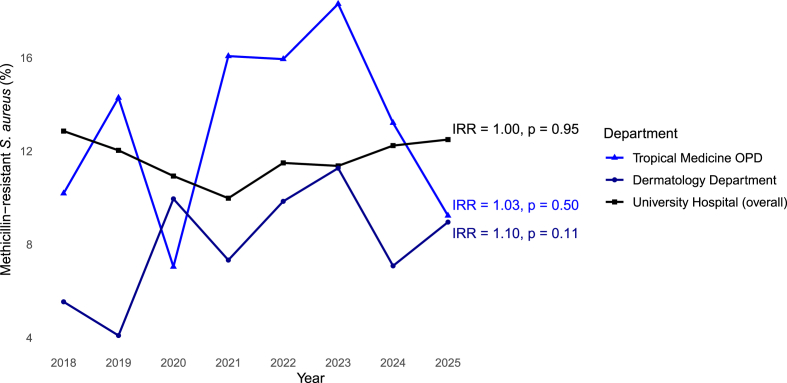
**Temporal trends in the percentage of MRSA among patients with *S. aureus* by clinical setting (2018**–**2025)** Annual percentage of patients with MRSA at the University Hospital in Berlin, stratified by clinical setting. IRRs and corresponding *p values* from regression models assessing temporal increases are displayed at the end of each series. Data for 2025 reflect the first half of the calendar year. Abbreviations: MRSA, methicillin-resistant *Staphylococcus aureus*; *S. aureus*, *Staphylococcus aureus*; OPD, outpatient department; IRRs, incidence risk ratios.

In total, 105/782 (13.4%) MRSA were seen at Tropical medicine OPD during the same time period. MRSA showed fluctuations with time, with yearly percentages ranging between 7% and 18.3%, but the rate of MRSA obtained from Poisson regression, did not increase significantly over time (IRR, 1.03; 95% CI, 0.95 – 1.12, *p = 0.50*); see Fig. 3. MRSA and Mup-RSA were seen in combination in 1/24 patients (4.2%) during the study period.

In a *post hoc* retrospective chart review of the 24 patients with a Mup-R positive sample evaluated at the Tropical Medicine outpatient Department, data from the standardised travel questionnaire that is filled out by all patients showed that the most commonly reported main region of exposure was Southeast Asia in 15/24 (62.5%), followed by 3/24 exposed in Latin America (12.5%), 3/24 in the Middle East (12.5%), and 2/24 (8.3%) in Sub-Saharan Africa. One patient had no history of travel outside of Germany within the past year. Most patients, 18/24 (75%), travelled for tourism, 1/24 (4.2%) travelled for work, and 4/24 (16.7%) were refugees with recent arrival in Germany. *S. aureus* was positive for the PVL toxin in 11 of the 23 patients who were tested (47.8%).

### 3.2 Department of dermatology

A total of 23/2030 (1.1%) Mup-RSA positive patients were observed during the full study period. The percentage of Mup-RSA–positive patients remained below 1% until 2021 but began to rise in 2022 (1.2%) and peaked in 2024 (3.1%), see Table 1 and Fig. 2. Each one-year increase corresponded to a 37% higher rate of Mup-R cases per patient with *S. aureus* isolated (IRR, 1.37; 95% CI, 1.12 – 1.67, *p < 0.01*) obtained from Poisson regression; Fig. 2.

In total, 157/2030 MRSA (7.7%) were seen during the same time period: the lowest percentage in MRSA in 2019 (4.1%) and the highest in 2023 (11.3%), but the rate of MRSA obtained from Poisson regression, did not increase significantly over time (IRR 1.10; 95% CI, 0.98 – 1.24, *p = 0.11*); see Fig. 3. Methicillin resistance in Mup-RSA was found in 1/23 patients (4.3%) during the study period.

### 3.3 University hospital overall

Mup-RSA was identified in a total of 142/28,122 patients (0.5%) during the study period, including the patients at Tropical Medicine OPD and Dermatology Department. The percentage of Mup-RSA remained stable and below 1% during the observation period (Table 1) and there was no significant increase in the Mup-R rate over time (IRR, 1.12; 95% CI, 0.99 – 1.26, *p = 0.09*) obtained from negative binomial regression; see Fig. 2.

A total of 3287/28,122 patients with MRSA out of all *S. aureus* positive patients (11.69%) were identified during the same time period. The overall MRSA percentages remained stable each year, with the lowest percentage observed in 2021 (10%) and the highest percentage in 2018 (12.9%), and, obtained from negative binomial regression, the rate of MRSA did not increase significantly over time (IRR, 1.0; 95% CI, 0.98 – 1.02, *p = 0.95*); see Fig. 3. Methicillin-resistance in Mup-RSA was found at the University hospital overall in 32/142 Mup-RSA positive patients (22.5%).

## 4 Discussion

At the Tropical Medicine OPD, Mup-RSA infections emerged in 2020 among returning travellers and migrants and have shown increasing rates thereafter, with the highest proportion observed in the first half of 2025 (9.2%). A less pronounced increase was observed in the Dermatology Department, but no clear increase was seen when assessing the University Hospital overall during the same time period. Of the 142 Mup-RSA positive patients identified across the University Hospital during the study period, 24 (16.9%) originated from the Tropical Medicine OPD and 23 (16.2%) from the Dermatology Department, even though these clinics accounted for only 2.8% and 7.2%, respectively, of all *S. aureus* positive patients. Thus, despite contributing just 10% of the overall *S. aureus* burden, the two clinics together accounted for approximately one third of all Mup-R cases, indicating a disproportionate concentration of Mup-R in these departments. These divergent patterns likely reflect the distinct epidemiological contexts represented by each setting. The Tropical Medicine OPD predominantly evaluates patients with imported purulent SSTIs caused by community-associated lineages, often PVL-positive, linked to exposure in regions outside Europe [14,26]. In contrast, the Dermatology Department primarily treats locally acquired infections. The overall data from the University Hospital encompasses a broad mixture of community- and hospital-associated lineages across diverse infection types, not limited to skin infections. Importantly heterogeneity in screening and diagnostic testing practices across departments and infection types should be considered when interpreting the overall yearly proportions. The Mup-R rate increase among imported infections at the Tropical Medicine OPD may reflect the introduction of resistant clones from high-prevalence regions (e.g., Asia) [27], while patterns in the Dermatology Department may mirror the circulation of local community clones.

The occurrence of Mup-R has been previously associated with patterns of mupirocin use, ranging from common resistance in settings with over-the-counter availability or widespread use in the general population, to rare or moderate resistance in places where use is limited, restricted to specific indications, or confined to short-term or targeted prophylactic applications [11,13]. Differences in regulatory control and prescribing practices across regions, including stricter prescription requirements in Germany, may partially explain variation in observed resistance. Apparent increases within the Tropical Medicine OPD subgroups may therefore arise from shifts in clone introduction, selective pressures, or both [10].

The setting-specific differences we observed are consistent with the broader literature, in which reported Mup-RSA prevalences vary widely depending on study period, geographical region, clinical setting, and antibiotic resistance thresholds used [27]. A 2019 systematic review reported a global rise in the prevalence of Mup-RSA, with pooled prevalences of 8.5% of high-level Mup-R among MSSA and MRSA isolates, the highest figures occurring in Asia and the Americas, and the lowest in Europe [27]. Only one study that included data from Germany in 2001 was identified [28]. In the only previous German study, high-level Mup-R occurred in 0.9% of *S. aureus* positive patients, and in 3.1% of MRSA positive patients [28]. Our analysis provides updated evidence from a German university hospital, showing that, overall, Mup-R remained uncommon, with annual rates below 1% and the majority of Mup-R isolates being MSSA. Of note, many previous studies report Mup-RSA only among MRSA isolates, which obscures the true prevalence and underestimates the burden in MSSA [[29], [30], [31], [32], [33], [34]]. In addition, MupRSA surveillance efforts may also be complicated by heterogeneity in laboratory reporting practices for mupirocin susceptibility, and varying definitions for resistance, with some studies reporting only high-level resistance and others including low-level resistance, limiting comparability [27]. We focused our analyses on high-level Mup-R (MIC >512 mg/L), corresponding to the EUCAST epidemiological cut-off used by the reference laboratory [12]. As a result, direct comparison with studies restricted to MRSA or those incorporating low-level resistance is not feasible. However, high-level resistance remains more relevant for clinical practice, as it has been clearly associated with adverse patient outcomes, including failure of mupirocin-based decolonisation strategies, while the role of low-level resistance remains unclear [11]. In addition, our broader approach of including patients with methicillin-sensitive *S. aureus* (MSSA) and MRSA provides a more complete picture of Mup-R, than only focusing on MRSA isolates.

Across the University hospital, MRSA proportions remained stable over time, without clear temporal increases. In the Dermatology Department, some year-to-year fluctuations were observed, but no consistent changes in proportions or trends emerged. In contrast, at Tropical Medicine OPD, MRSA showed an upward pattern beginning in 2020, with a peak exceeding 20% in 2023, but no statistically significant change.

MRSA with concurrent Mup-R was identified in only a single patient at the Tropical Medicine OPD and in only one sample from the Dermatology Department. This observation differs from a previous German study, where most Mup-R resistance was seen in patients with MRSA [28]. Of note, almost 50% of the patients from the Tropical Medicine OPD were positive for PVL. This finding may indicate that Mup-RSA isolates identified in returning travellers and migrants represent distinct community-associated lineages, including PVL-positive MSSA clones circulating in tropical and subtropical regions, rather than the healthcare-associated clones more commonly observed in the German hospital setting. This would support the hypothesis that imported and locally acquired infections involve different *S. aureus* lineages and highlighting the need for continued surveillance.

In a *post hoc* review of exposure history, most patients with Mup-R evaluated at the tropical medicine OPD reported exposure in Southeast Asia. However, as this is also a frequent travel destination among patients presenting to our clinic, this finding should be interpreted cautiously and warrants further investigation, including molecular characterization.

Our findings should be viewed within the broader context of antimicrobial resistance (AMR), which continues to escalate worldwide. AMR is recognized as one of the top ten global health threats, largely driven by the misuse and overuse of antimicrobials [35]. Travel and migration facilitate the dissemination of resistant organisms, with skin infections constituting a frequent and clinically relevant manifestation and the leading dermatological diagnoses among travellers returning from tropical and subtropical regions [4,14,26,36,37]. Besides *S. aureus*, travel has similarly been implicated in the acquisition and importation of other AMR pathogens, including ESBL-producing *Enterobacteriaceae*, supporting the concept that international travel contributes to the cross-border dissemination of AMR [38].

Notwithstanding its relevance, awareness of Mup-R remains low, and its clinical significance is not well recognized; however, Mup-R could be a significant factor for failure of decolonisation efforts for PVL or MRSA [11]. These gaps in awareness are compounded by the limited therapeutic options currently available. In cases of Mup-R, alternative antiseptic agents (e.g., polyhexanide, octenidine) may be considered. However, because they act nonspecifically rather than targeting *S. aureus* bacteria, their ability to achieve effective eradication compared to mupirocin seems to be limited [11,39]. A 2015 review identified a lack of head-to-head trials comparing alternative decolonisation regimens with the standard combination of mupirocin and chlorhexidine. Among the few comparative studies, a polyhexanide-based regimen showed lower decolonisation success (29%) compared to mupirocin and chlorhexidine [11]. More than a decade later, however, there appears to be limited additional comparative research. Evidence on polyhexanide-based decolonisation strategies, to our knowledge, remains scarce. In a randomised, double-blind, placebo-controlled trial with a median of nine days of therapy, polyhexanide did not significantly improve eradication rates compared with placebo (33.8% vs. 29.3%) [40]. A single-centre, non-randomised open-label trial found that an integrated polyhexanide-based decolonisation strategy continued after hospital discharge achieved higher overall decolonisation rates than inpatient-only polyhexanide-based treatment (47% vs. 12%) [41]. This suggests that when using polyhexanide-based products, longer treatment durations may improve effectiveness, although overall success rates remain modest. Despite these limitations, polyhexanide is currently among the few available topical options and is therefore relied upon in clinical practice [11]. Additional candidates, including tea tree oil, bacteriophages, and other natural antiseptic or antibacterial agents, require further evaluation [11]. In a more recent randomized controlled trial comparing medical-grade honey with intranasal mupirocin for MRSA nasal decolonisation, eradication rates were lower with honey (42.8% vs. 56.8%), but the difference was not statistically significant [42]. Our study is limited by its retrospective design, since microbiological testing occurred at clinicians' discretion, some patients may not have been tested and instead treated empirically. Such selective sampling could under- or overestimate resistance and influence observed trends, particularly if testing thresholds varied over time. Additionally, our centre's reference laboratory reports data on high-level Mup-R, but not on low-level resistance, and, even though the clinical importance of low-level resistance remains unclear [11,43], a case control study found that low-level Mup-R, accompanied by genotypic chlorhexidine resistance, was a risk factor for decolonisation failure [5]. This may be relevant to clinical practice because mupirocin and chlorhexidine are frequently administered together as part of decolonisation regimens and may explain treatment failure in patients without high-level Mup-R. In addition to methicillin resistance, we did not assess co-resistance to other antimicrobial classes, although high-level mupirocin resistance may be associated with increased resistance to other antibiotics [11].

Our study has several strengths. It draws on systematically collected laboratory data over multiple years and combines institution-wide surveillance with data stratified by two departments, one of which tailors to patients with imported infections. Furthermore, routine reporting of mupirocin susceptibility at our institution enabled early detection of this emerging and increasing resistance rate. Overall, we provide insight into the emergence of imported Mup-RSA and highlight the need for continued surveillance and clinical awareness. Although no clear increase in local Mup-R rates was observed hospital-wide, rising case numbers in the Tropical Medicine and Dermatology departments indicate a need for ongoing clinical and epidemiologic surveillance to detect potential future spread, particularly in settings where mupirocin use is common. Ongoing collaborative clinical and molecular surveillance are needed to monitor resistance patterns over time and to clarify the clinical importance of Mup-R across settings, particularly in returning traveller clinics, where early signals may foreshadow broader local dissemination.

## 5 Conclusion

From 2018 to the first half of 2025, Mup-RSA rose predominantly in patients attending the Tropical Medicine OPD, with more modest increases in patients attending the Dermatology Department, while the overall rate at the University hospital remained stable. Clinicians should be aware of Mup-RSA emergence and of the availability of alternative disinfectant nasal ointments. Mupirocin susceptibility testing may be warranted in patients with indications for decolonisation (e.g., MRSA, PVL), especially in those with a history of travel or previous decolonisation failure. Future research should focus on molecular surveillance of clonal lineages, risk of mupirocin use patterns on Mup-R development, assessment of antibiotic co-resistances, and on clinical implications, such as nasal decolonisation failure in patients with MRSA, and/or PVL-positive strains.

## CRediT authorship contribution statement

**Maria Cristina Moreno-del Castillo:** Writing – original draft, Methodology, Investigation, Formal analysis, Data curation, Conceptualization. **Daniel Humme:** Writing – review & editing, Investigation, Data curation. **Rasmus Leistner:** Writing – review & editing, Investigation. **Renate Krüger:** Writing – review & editing, Investigation. **Miriam Stegemann:** Writing – review & editing, Investigation. **Leif Hanitsch:** Writing – review & editing, Investigation. **Franziska Layer-Nicolaou:** Writing – review & editing, Investigation. **Andreas Knaust:** Writing – review & editing, Data curation. **Josef G. Sägmüller:** Writing – review & editing, Data curation. **Angela Stein:** Writing – review & editing, Data curation. **Paul Pitzinger:** Writing – review & editing, Investigation. **Alice Hucko:** Writing – review & editing, Investigation. **Jessica L. Rohmann:** Writing – review & editing, Methodology, Formal analysis. **Frank P. Mockenhaupt:** Writing – review & editing, Supervision, Project administration. **Beate Kampmann:** Writing – review & editing, Supervision, Project administration. **Dennis Nurjadi:** Writing – review & editing. **Philipp Zanger:** Writing – review & editing, Investigation. **Andreas K. Lindner:** Writing – original draft, Supervision, Project administration, Methodology, Investigation, Conceptualization.

## Data availability statement

Due to the sensitive nature of the clinical data and applicable data protection regulations, as well as the small patient numbers and risk of re-identification, the datasets are not publicly available. De-identified data may be shared with qualified researchers upon reasonable request to the corresponding author, and subject to ethical approval.

## Declaration of generative AI and AI-assisted technologies in the writing process

During the preparation of this work, the authors used ChatGPT 5.1 to provide writing assistance, specifically to improve clarity, grammar, and consistency of phrasing. R code development and completion were assisted by GitHub Copilot (GitHub, San Francisco, CA). The AI tools were not used for methodological decisions, interpretation of findings, or as a substitute for scientific reasoning. The authors reviewed and edited the content as needed and take full responsibility for the content of the published article.

## Study funding

This research did not receive any specific grant from funding agencies in the public, commercial, or not-for-profit sectors.

## Declaration of competing interest

The authors declare that they have no known competing financial interests or personal relationships that could have appeared to influence the work reported in this paper.
